# Purification and Structural Characterization of Siderophore (Corynebactin) from *Corynebacterium diphtheriae*

**DOI:** 10.1371/journal.pone.0034591

**Published:** 2012-04-13

**Authors:** Sheryl Zajdowicz, Jon C. Haller, Amy E. Krafft, Steve W. Hunsucker, Colin T. Mant, Mark W. Duncan, Robert S. Hodges, David N. M. Jones, Randall K. Holmes

**Affiliations:** 1 Department of Microbiology, University of Colorado School of Medicine, Aurora, Colorado, United States of America; 2 Department of Biology, Metropolitan State College of Denver, Denver, Colorado, United States of America; 3 Respiratory Diseases Branch/Division of Microbiology and Infections Diseases/National Institute of Allergy and Infectious Diseases/National Institutes of Health/Department of Health and Human Services, Bethesda, Maryland, United States of America; 4 Department of Endocrinology, Metabolism, and Diabetes, University of Colorado School of Medicine, Aurora, Colorado, United States of America; 5 Department of Biochemistry and Molecular Genetics, University of Colorado School of Medicine, Aurora, Colorado, United States of America; 6 Department of Pharmacology, University of Colorado School of Medicine, Aurora, Colorado, United States of America; East Carolina University School of Medicine, United States of America

## Abstract

During infection, *Corynebacterium diphtheriae* must compete with host iron-sequestering mechanisms for iron. *C. diphtheriae* can acquire iron by a siderophore-dependent iron-uptake pathway, by uptake and degradation of heme, or both. Previous studies showed that production of siderophore (corynebactin) by *C. diphtheriae* is repressed under high-iron growth conditions by the iron-activated diphtheria toxin repressor (DtxR) and that partially purified corynebactin fails to react in chemical assays for catecholate or hydroxamate compounds. In this study, we purified corynebactin from supernatants of low-iron cultures of the siderophore-overproducing, DtxR-negative mutant strain *C. diphtheriae* C7(β) Δ*dtxR* by sequential anion-exchange chromatography on AG1-X2 and Source 15Q resins, followed by reverse-phase high-performance liquid chromatography (RP-HPLC) on Zorbax C8 resin. The Chrome Azurol S (CAS) chemical assay for siderophores was used to detect and measure corynebactin during purification, and the biological activity of purified corynebactin was shown by its ability to promote growth and iron uptake in siderophore-deficient mutant strains of *C. diphtheriae* under iron-limiting conditions. Mass spectrometry and NMR analysis demonstrated that corynebactin has a novel structure, consisting of a central lysine residue linked through its α- and ε- amino groups by amide bonds to the terminal carboxyl groups of two different citrate residues. Corynebactin from *C. diphtheriae* is structurally related to staphyloferrin A from *Staphylococcus aureus* and rhizoferrin from *Rhizopus microsporus* in which d-ornithine or 1,4-diaminobutane, respectively, replaces the central lysine residue that is present in corynebactin.

## Introduction

Iron is essential for growth of most bacteria. Although iron is plentiful in animals and humans, most of it is complexed with iron-binding proteins such as transferrin, lactoferrin, or ferritin, or incorporated into compounds such as heme, or hemoglobin. To overcome the limited bioavailability of iron from such sources, most pathogenic bacteria, including *Corynebacterium diphtheriae*, use energy-dependent iron acquisition systems to assimilate iron from their hosts [1], [2], [3], [4]. One class of iron acquisition systems uses siderophores, low-molecular-weight iron-chelating compounds that are secreted by bacteria under low-iron growth conditions, to scavenge host iron. After sequestration of free iron or removal of iron from host iron-binding proteins, the iron-siderophore complexes bind to specific receptors at the bacterial cell surface and are transported into the bacterium where the iron is released either by reduction of the iron [2] or by degradation of the siderophore [5].

Siderophores are a structurally diverse group. Siderophores can be classified by their metal binding groups as catecholates, hydroxamates, or complexones (a heterogenous group with other metal-binding ligands [6], [7]. Enterobactin, the prototype for catecholate siderophores, is a trimeric cyclic ester consisting of 2,3-dihydroxybenzoyl-serine subunits [7]. Ferrichrome is typical of hydroxamate siderophores, which contain either *N* ^6^-acyl-*N* ^6^-hydroxylysine or *N* ^5^-acyl-*N* ^5^-hydroxyornithine [7]. Staphyloferrin A and rhizoferrin represent a subgroup of complexone siderophores containing two citric acid subunits, with the central ∝-hydroxycarboxylic acid moiety of each citrate serving as an iron-complexing ligand [8], [9], [10], [11], [12], [13].

In the mid 1980s, Russell and colleagues discovered the siderophore from *C. diphtheriae* strains C7 and C7(β), partially purified it, and named it corynebactin [14], [15], [16]. The partially purified siderophore failed to react in assays for catecholate and hydroxamate groups. Furthermore, the partially purified siderophore (or supernatants from low-iron cultures of wild type *C. diphtheriae*) promoted growth and iron-uptake in *C. diphtheriae* C7(β) strain HC6 (a chemically-induced siderophore-deficient mutant of *C. diphtheriae* C7(β) [14]) and the Park-Williams 8 strain of *C. diphtheriae* (which produces large amounts of diphtheria toxin,is used for commercial production of diphtheria toxoid for vaccine, and is also deficient in siderophore production [16], [17]). In 2005, Kunkle and Schmitt identified the *ciuA-G* gene cluster in *C. diphtheriae*, showed that the *ciuA-D* operon (which has predicted products similar to ABC-type transporters) is essential for siderophore-dependent iron uptake, demonstrated that the *ciuE* gene is required for biosynthesis of siderophore, and constructed deletion mutations in the *ciuE* genes of the *C. diphtheriae* laboratory reference strain C7(−) and the *C. diphtheriae* clinical isolate strain 1737 [18]. Kunkle and Schmitt noted that *ciuF* has weak similarity to some membrane efflux proteins from gram-positive bacteria whereas *ciuG* has no similarity to other known proteins; they did not investigate experimentally possible roles for *ciuF* or *ciuG* in siderophore production or siderophore-dependent iron uptake in *C. diphtheriae* [18].

In 1997, Budzikiewicz *et al.* reported that *Corynebacterium glutamicum* produces a novel catecholate siderophore (a trimeric cyclic ester consisting of 2,3-dihydoxybenzoyl glycylthreonine subunits) which they also named corynebactin [19]. In 2001, May *et al.* [20] showed that the *dhb* operon of *Bacillus subtilis* controls biosynthesis of a catecholate siderophore with a structure identical to that described by Budzikiewicz et al [19], which they named bacillibactin. In 2006, Dertz *et al.* performed comparative studies on siderophore-dependent iron uptake in *B. subtilis* and *C. glutamicum* [21]. They showed *C. glutamicum* does not produce a siderophore that can be detected by the Chrome Azural S (CAS) assay, nor can it take up iron from ferric bacillibactin. In addition, their analysis of the published genome sequence of *C. glutamicum* [22],[23] showed that it lacks homologs of the genes required for bacillibactin biosynthesis. Based on these findings, Dertz *et al* [21] recommended that the name bacillibactin be used for the trimeric catecholamide siderophore from *B. subtilis* described above and that the name corynebactin be reserved, consistent with historical precedent, for the siderophore from *C. diphtheriae*. Consistent with their recommendation, we will continue to use the name corynebactin for the siderophore from *C. diphtheriae*.

In the current study, we purified corynebactin from *C. diphtheriae*, determined its structure, and demonstrated that purified corynebactin has biological activity as a siderophore in *C. diphtheriae*.

## Results

### Purification and biological activity of corynebactin

Previous work in our laboratory achieved partial purification of corynebactin from *C. diphtheriae* [14]. In the current study, we developed methods to purify corynebactin from *C. diphtheriae* strain C7(β) Δ*dtxR*, a mutant that overproduces the siderophore [24], by use of sequential anion exchange chromatography and C8 reverse-phase high-performance liquid chromatography (RP-HPLC). We have performed multiple purifications of corynebactin and the following data, from a single purification experiment, are representative of all of these.

Pooled supernatants from cultures grown under low-iron conditions were sterilized by filtration, applied to an AG1X-2 anion exchange column, and eluted step-wise with 2.5% and 6% ammonium acetate. Preliminary experiments showed that most of the corynebactin detected by the CAS assay was present in the 6% ammonium acetate eluate (data not shown) and that an additional step-wise elution with 8.5% ammonium acetate yielded little additional siderophore. The 6% ammonium acetate eluate was lyophilized. The dried material was dissolved in water, applied to a Source15Q anion exchange column, and eluted with a linear 2.5%–7.5% gradient of ammonium acetate at pH 6.5 (Fig. S1). Quantitative CAS assays showed that corynebactin eluted as a single peak, centered at 6% ammonium acetate. The fractions containing corynebactin were pooled and lyophilized.

**Figure 1:**
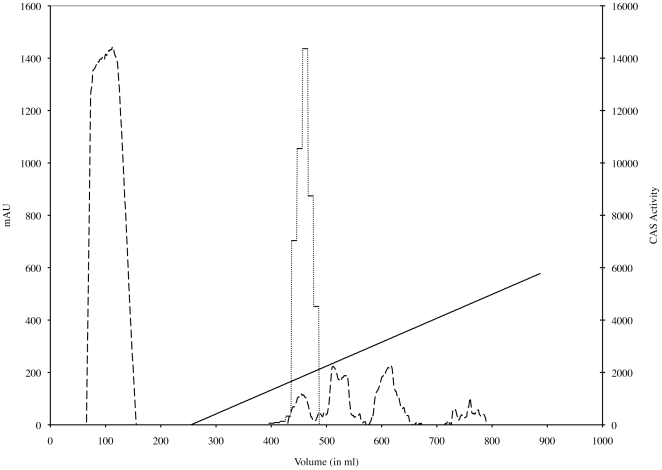
Final purification of corynebactin by C8 reversed-phase HPLC. Corynebactin was recovered as a single activity peak coincident with an A_210_ absorbance peak at approximately 9.4% acetonitrile during elution with a 0% to 80% linear gradient of acetonitrile in 0.6% formic acid. A_210_ is shown as a dashed line; the linear acetonitrile gradient is shown as a solid line; and CAS activity for each fraction is shown as a dotted line.

In the final purification step, the lyophilized material was dissolved in water, adjusted to pH 3.1 by drop-wise addition of formic acid, loaded onto a C8-RP-HPLC column equilibrated with 0.6% formic acid, and eluted with a linear gradient from Buffer A (0.6% formic acid) to Buffer B (0.6% formic acid, 80% acetonitrile) as shown in Fig. 1. Quantitative CAS assays showed that corynebactin eluted as a single peak that was centered at 9.5% acetonitrile and was coincident with a small 210 nm absorbance peak. The overall yield of purified corynebactin was about 38% of the siderophore activity detected by the CAS assay in the original culture supernatant, and the iron-binding activity in the CAS assay of the purified corynebactin was equivalent to about 127 µmoles of EDDA.

**Figure 2:**
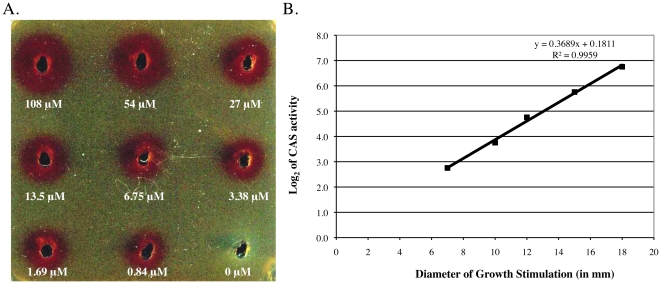
Quantitative bioassay for corynebactin. A. Varying concentrations of corynebactin (expressed as EDDA equivalents) were added to wells containing 25 µM FeCl_3_. Growth of *C. diphtheriae* C7(β) Δ*ciuE* was stimulated by corynebactin, and the growing bacteria reduced the triphenyltetrazolium chloride indicator dye and turned red. No visible bacterial growth was present around the control well without corynebactin. The figure shows the results of a representative bioassay. B. Within the range from 6.75 µM to 108 µM EDDA equivalents, the average diameter of the growth stimulation zone for *C. diphtheriae* C7(β) Δ*ciuE*, based on triplicate samples, was directly proportional to the log_2_ of the concentration of the corynebactin sample in the well.

**Figure 3:**
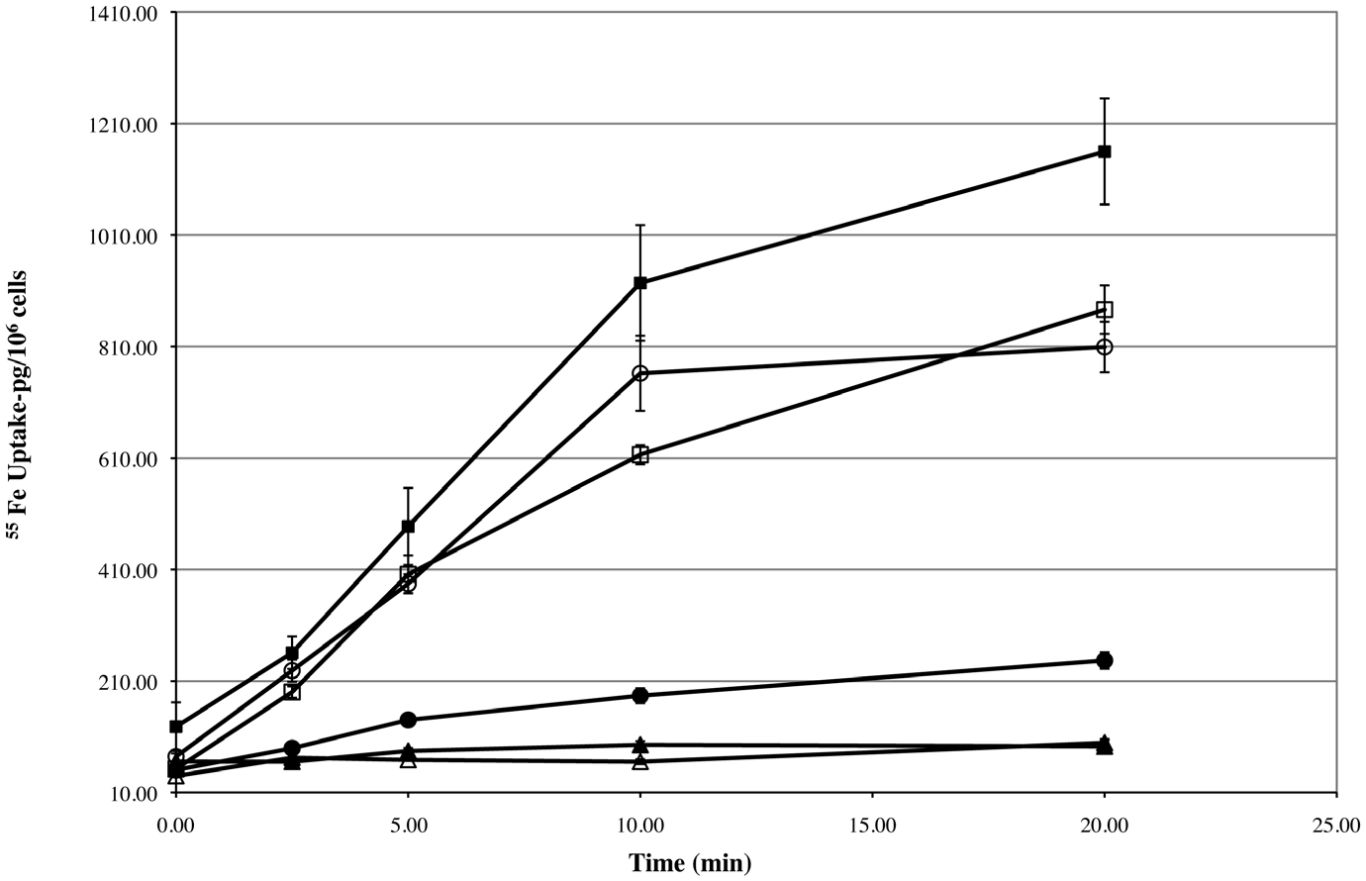
Effects of purified corynebactin on uptake of ^55^Fe by wild-type and mutant strains of *C. diphtheriae*. The strains of *C. diphtheriae* tested are as follows: wild type C7(β) (▪, □); corynebactin production deficient C7(−) Δ*ciuE* (•, ○); and corynebactin utilization deficient C7(β) Δ*ciuA* (▴, ▵). Filled symbols show the results of assays performed in medium without added corynebactin, and open symbols show the results of assays performed in medium with addition of purified corynebactin at 4 µM.

The biological activity of corynebactin in supernatants from low-iron cultures of *C. diphtheriae* or in partially purified preparations was demonstrated in previous studies by either: a) stimulation of growth of corynebactin-production-deficient strains of *C. diphtheriae* in liquid cultures or plate assays, or b) by stimulation of uptake of ^59^Fe^3+^ by washed cells of wild-type or corynebactin-production-deficient mutant strains of *C. diphtheriae* [16], [17], [18]. In this study, we modified a previously described agar diffusion bioassay for corynebactin [18] by adding an agarose overlay containing triphenyltetrazolium chloride to the bioassay plates. When corynebactin was added to wells cut into the bioassay plates, the growing *C. diphtheriae* C7(−) Δ*ciuE* indicator bacteria reduced the triphenyltetrazolium chloride and turned red, thereby making it easier to see and measure the diameters of the growth stimulation zones surrounding the wells (Fig. 2A). Growth stimulation of the *C. diphtheriae* C7(−) Δ*ciuE* indicator bacteria in the plate bioassays was more robust and easier to measure when corynebactin was added to the wells together with 25 µM Fe^3+^ (Fig. 2A) instead of without added iron (data not shown). Within the range from 6.75 µM to 108 µM, the average diameter of the growth stimulation zone was directly proportional to the log_2_ of the corynebactin concentration (Fig. 2B). The agar diffusion bioassay described here can therefore be used to measure corynebactin activity quantitatively in experimental samples by comparing their potency to that of reference samples of purified corynebactin within the linear range of the dose-response curve. In addition, we performed direct assays to determine the ability of purified corynebactin to stimulate ^55^Fe^3+^ uptake by wild-type *C. diphtheriae* C7(β), by the corynebactin-production-deficient *C. diphtheriae* C7(−) Δ*ciuE* mutant, and by the corynebactin-utilization-deficient *C. diphtheriae* C7(β) Δ*ciuA* mutant (Fig. 3). These experiments demonstrated directly that purified corynebactin can stimulate iron uptake by the corynebactin-production-deficient C7(−) Δ*ciuE* mutant but has little effect on either the ability of the wild type C7(β) strain or the inability of a corynebactin-utilization-deficient C7(β) Δ*ciuA* mutant to take up iron [14], [18]. Taken together, our findings show that corynebactin, after purification to apparent homogeneity from low-iron culture supernatants of *C. diphtheriae* C7(β) Δ*dtxR*, exhibits the biological activities previously attributed to *C. diphtheriae* siderophore based on studies with low-iron culture supernatants or partially purified preparations of siderophore.

### Chemical analysis of purified corynebactin

We confirmed that purified corynebactin gave negative reactions in the Arnow test for catecholates and the Csaky test for hydroxamates, as reported previously for partially purified corynebactin [14]. Because many siderophores that lack catecholate or hydroxamate moieties contain citrate [7], we tested an acid hydrolysate of purified corynebactin in a quantitative bioassay and demonstrated the presence of citric acid (data not shown). We also demonstrated the presence of lysine in the hydrolysate by amino acid analysis (data not shown). Based on the results of these assays, we estimated that the stoichiometric ratio of citric acid to lysine was approximately 2∶1, but these tests did not exclude the possible presence of other components in corynebactin.

### Determination of corynebactin structure by NMR spectroscopy

**Figure 4:**
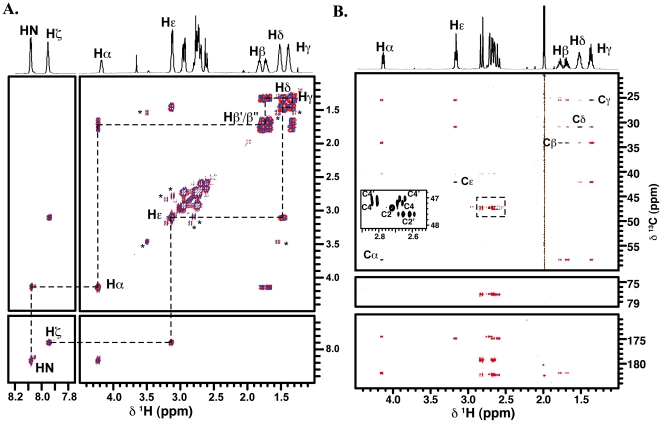
Analysis of corynebactin structure by NMR spectroscopy. A. ^1^H-^1^H DQF-COSY spectrum of corynebactin dissolved in 90% H_2_O at pH 4.0. Dashed lines illustrate the correlations between the protons in the central lysine core moiety B. Overlay of the ^1^H-^13^C HSQC Spectrum of corynebactin dissolved in D_2_O at pH 6.0 (black) with the ^1^H-^13^C HMBC (red). The inset shows an expansion of the region indicated by the dashed box.

A one dimensional (1D) ^1^H NMR spectrum of a sample of purified corynebactin dissolved in 90% H_2_O/10% D_2_O was used as the basis for spectral analysis. From the final analysis we propose that the structure consists of a single central lysine substituted at both nitrogen atoms with citric acid molecules. Key aspects in this structure determination involved the two protons at 7.94 and 7.86 ppm (Fig. 4A). In a double quantum filtered correlated spectroscopy (DQF-COSY) spectrum these peaks showed scalar coupling to peaks at 4.15 and 3.17 ppm respectively (Fig. 4A) but showed no correlations to any carbon atoms in a ^1^H-^13^C Heteronuclear Single Quantum Correlation (HSQC) spectrum (black peaks in Fig. 4B). Further, these peaks are absent in spectra recorded using samples dissolved in 100% D_2_O indicating that they are likely to originate from slowly exchanging amide protons. This was subsequently confirmed using a ^1^H-^15^ N HSQC spectrum (data not shown) that established ^15^N chemical shifts for these amide groups at 135.2 and 131.1 ppm respectively.

**Figure 5:**
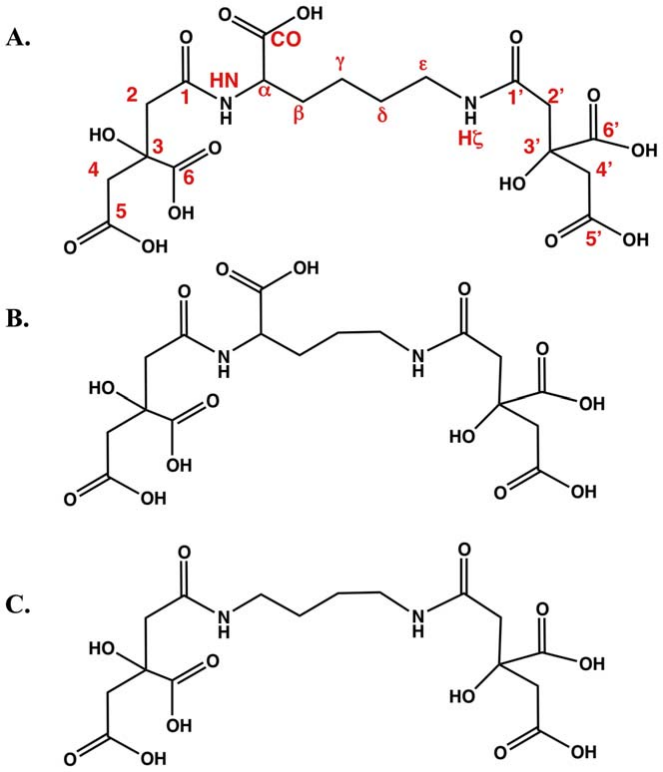
Comparison of structures of corynebactin, staphyloferrin A, and rhizoferrin. A. Structure of corynebactin determined by NMR spectroscopy. B. The published structure of staphyloferrin. C. The published structure of rhizoferrin.

The DQF-COSY spectrum established that these two amide protons were located at opposite ends of an isolated spin-system consistent with the presence of a lysine core. This was confirmed using a ^1^H-^13^C HSQC experiment to make assignments of the directly attached carbons, and a long-range ^1^H-^13^C Heteronuclear Multiple Bond Correlation (HMBC) experiment to establish connections to neighboring carbons. These experiments establish that the proton at 7.93 ppm originates from the backbone HN of the lysine, as the adjacent Hα and Hβ protons are correlated to the same carbonyl group at 181.9 ppm (labeled CO in Fig. 5A), consistent with the presence of carboxylic acid group in an amino acid. Additionally, the Hα proton shows a correlation to a second carbonyl group at 174.5 ppm (subsequently assigned to C1 in Fig. 5A), consistent with formation of an amide bond with the main chain nitrogen. In contrast the Hε group is correlated to only a single carbonyl at 174.8 ppm (group C1′) again indicative of formation of an amide bond.

In the ^1^H-^13^C HSQC there were no C-H groups with ^13^C chemical shifts above 60 ppm (Fig. 5B). This spectrum revealed that the group of peaks observed in the range of 2.6–2.8 ppm in the ^1^H NMR spectrum (which integrated for 8 protons) resolved into four isolated CH_2_ groups, which are shown as an inset in Fig. 4B. These methylene groups show no correlations to other protons within the structure in the DQF-COSY except to their geminal proton partner (Fig. 4A). However, all of these methylene groups show a number of long-range correlations in the HMBC (Fig. 5A). This experiment established that two of theses groups (subsequently labeled C2′ and C4′) are correlated to each other through a quaternary center at 77.5 ppm (group C3′), and share a correlation to carbonyl group at 182.3 ppm (C6′). We found that the C2′ methylene shares a correlation with Hε of the central lysine to the C1′-carbonyl at 174.8 ppm, and so this establishes the orientation of the spin system that substitutes to the lysine. Finally, the C4′-methylene is correlated to another carbonyl at 179.2 ppm (C5′). The pattern of adduction with the lysine residues was further conformed by observation of through space ^1^H-^1^H NOEs from the Hζ amide to the Hε protons and to the C2′ methylene protons in a NOESY spectrum recorded in 90% H_2_O/10% D_2_O (data not shown).

A similar pattern of correlations was observed from the methylene groups subsequently assigned to C2 and C4, but in this case the HMBC and NOESY spectra establish that these methylenes are part of a substitution that occurs to the main chain amide (HN) of the lysine moiety. Complete chemical shift assignments are presented in Table 1.

**Table 1:** List of Chemical Shifts for Corynebactin.

Group	^1^H (ppm)	^13^C (ppm)	^15^N (ppm)
*Lysine*			
HN	7.93		135.2
α	4.15	57.84	
β	1.77, 1.68	34.11	
γ	1.35	25.45	
δ	1.51	30.87	
ε	3.17	42.06	
ζ	7.85		131.1
CO		181.9	
*Citrate 1 (HN adduct*)			
C1		174.5	
C2	2.72	47.34	
C3		77.41	
C4	2.82, 2.67	47.17	
C5		179.2	
C6		182.1	
*Citrate 2 (H*ζ *adduct)*			
C1′		174.8	
C2′	2.67, 2.60	47.56	
C3′		77.51	
C4′	2.83, 2.66	47.0	
C5′		179.2	
C6′		182.3	

The group assignment is as presented in Fig. 5-C. All shifts are referenced to internal DSS.

Based on the analysis of the HSQC, HMBC, DQF-COSY and NOESY experiments we propose the structure shown in Fig. 5A, in which a lysine amino acid is substituted at both amino groups with two molecules of citrate. The full set of chemical shifts is presented in Table 1. The structure of corynebactin is very similar to the structures of staphyloferrin A [12] (Fig. 5B), in which an ornithine molecule is adducted with two citrate molecules through the two amino groups, and rhizoferrin [12]] (Fig. 5C), in which the central lysine residue is replaced by 1,4-diaminobutane. This structure fully explains all the observed correlations in the NMR experiments. The mass predicted by this structure was confirmed by mass spectrometry of the purified corynebactin.

### Electrospray ionization mass spectrometry

**Figure 6:**
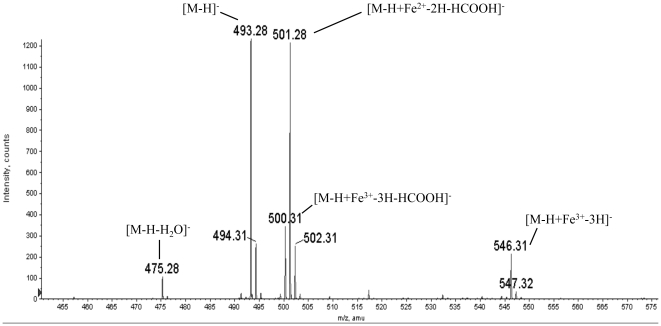
Analysis of corynebactin by electrospray ionization mass spectra (negative ion mode). A. A representative mass spectrum for corynebactin. The average *m/z* value for corynebactin was 493.138 based on the average of five separate mass spectra. Additional peaks at 475.28, 500.31, 501. 28, and 546.31 correspond to the following ions: [M-H-H_2_O]^−^, [M-H+Fe^2+^-2H-HCOOH]^−^, [M-H+Fe^3+^-3H-HCOOH]^−^, and [M-H+Fe^3+^-3H]^−^, respectively.

Based on the proposed structure for corynebactin deduced from our NMR data and the structures of the closely related siderophores staphyloferrin A [12]and rhizoferrin [11]characterized previously, we assumed the compound to be anionic. Therefore, we acquired the mass spectrum of purified corynebactin in negative ion mode. A representative negative ion mass spectrum is shown in Fig. 6. One dominant peak (*m/z* 493.28) has a mass consistent with the structure proposed following NMR analysis (*i.e*., [M-H]^−^) Based on five separate determinations., the average measured value for this dominant peak (*m/z* 493.138) is within 20 ppm of the calculated mass (*m/z* 493.131) of the molecular anion of the proposed structure for corynebactin shown in Fig. 5A. Siderophores can bind ferric irons with very high affinity, and the peak (*m/z* 546.31) corresponds to the ion [M-H+Fe^3+^-3H]^−^ (e.g., the molecular anion coordinated to Fe^3+^ with the corresponding loss of three protons to give a single negative charge on the ion). Electrospray ionization mass spectrometry typically results in the loss of neutral molecules from the protonated or deprotonated molecule. Two peaks (*m/z* 475.28) and (*m/z* 500.31) correspond to the loss of water from the deprotonated molecule [M-H-H_2_0]^−^ and loss of formic acid from the deprotonated iron complex [M-H+Fe^3^-3H-HCOOH]^−^, respectively. The peak at *m/z* 501.28 is consistent with the proposed structure [M-H+Fe^2+^-2H-HCOOH]^−^. We suggest that this ion arises from the peak at *m/z 500.31* by reduction of iron from Fe^3+^ to Fe^2+^ and the concurrent addition of a proton. The peaks at 494.31, 502.31 and 547.32 are components of the isotopic envelopes for the ions at 493.28, 501.28 and 546.31, respectively. Because corynebactin appears as a single peak when subjected to multiple off-line HPLC runs, we assume it to be a single species with the structure shown in Fig. 5A. All of the ions seen in the spectrum in Fig. 6 are consistent with this interpretation (*i.e.*, either with or without the molecule coordinated to iron). It is also important to note that the intensities of the individual *m/z* peaks in the spectrum (Fig. 6) do not necessarily reflect the relative abundances of the corresponding species in the original corynebactin sample. Iron may be picked up, lost, or reduced at various stages during liquid chromatography and electrospray ionization.

## Discussion

Many bacterial pathogens secrete high-affinity, low molecular weight siderophores to scavenge the iron that is essential for their survival. The siderophores are classified into three major categories based on their functional metal binding groups: i.e., catecholates, hydroxamates, and a heterogenous group called complexones. Here, we purified and characterized corynebactin from *C. diphtheriae* and showed that it has a novel structure. Specifically, we demonstrate that corynebactin is polycarboxylate in nature, consists of two citrates adducted to a lysine backbone, and has a molecular mass of 493.131. Also, as previously suggested by Russell et al. [14], based on studies with partially purified corynebactin from *C. diphtheriae*, the purified compound is able to chelate iron and promote the active transport of iron into *C. diphtheriae*. Furthermore, using purified corynebactin, we confirmed the finding reported originally by Kunkle and Schmitt [18] that corynebactin-dependent transport of ferric iron into *C. diphtheriae* is mediated by the specific receptor CiuA.

Structural analysis indicates that corynebactin falls into the heterogeneous group of siderophores called complexones and is structurally related to staphyloferrin A [12], [25] and rhizoferrin [10], [11], [26]. Staphyloferrin A, a siderophore produced by *Staphylococcus aureus*, consists of two citrate moieties linked via amide bonds to an ornithine backbone [12]; by contrast, rhizoferrin, a siderophore produced by both fungal and bacterial species, has two citrate moieties linked via amide bonds to a putrescine backbone [26]. The mass spectra of corynebactin and rhizoferrin are strikingly similar and provide further evidence for similarities between these two siderophores.

There are two distinct types of biosynthetic pathways for siderophores. The first is the nonribosomal peptide synthesis (NRPS)-dependent pathway that uses modular multi-functional enzymes to produce siderophores such as enterobactin [27] and yersiniabactin [28]. The second is the nonribosomal peptide synthesis-independent siderophore (NIS) biosynthetic pathway that produces siderophores such as aerobactin [29], rhizobactin [30], achromobactin [31], staphyloferrin A [32], and staphyloferrin B [33]. Kunkle and Schmitt [18] identified and characterized a gene cluster (*ciuABCDEFG*) in *C. diphtheriae* that is involved in import, biosynthesis, and putative export of corynebactin. They showed that CiuE, which is required for production of corynebactin, consists of homologous N- and C-terminal regions, both of which exhibit a comparable degree of relatedness to the IucA and IucC NIS synthetases involved in aerobactin biosynthesis. IucA catalyzes formation of an amide bond between a prochiral carboxyl group of citrate and the alpha amino group of N6-acetyl-N6-hydroxylysine, and IucC completes the synthesis of aerobactin by catalyzing formation of a second amide bond between the terminal carboxyl group of the previous adduct and a second molecule of N6-acetyl-N6-hydroxylysine [34]. The structure that we determined for corynebactin, consisting of a lysine residue linked through both of its amino groups via amide bonds to two molecules of citrate is therefore consistent with a biosynthetic pathway mediated by NIS synthetases. Additional studies will be required to show whether the homologous N- and C-terminal regions of CiuE both have enzymatic activity, and whether each has a specific role in biosynthesis of corynebactin.

More recently, Beasley et al. [32], characterized the locus that determines production of staphyloferrin A in *S. aureus* and identified two genes (*sfaD* and *sfaB*) within the locus that encode predicted proteins homologous to IucA and IucC. Cotton et al [35] showed that these two proteins (which they called SfnaB and SfnaD) catalyze the formation of staphyloferrin A *in vitro* when they are incubated with citrate, d-ornithine, and ATP. Their study showed that both SfnaB and SfnaD are required for synthesis of staphyloferrin A, that SfnaD catalyzes an initial condensation of citrate with d-ornithine, and that SfnaB acts on the resulting δ-citryl-d-ornithine adduct to complete the biosynthesis of staphyloferrin A [35]. It is reasonable to propose that the N- and C-terminal regions of CiuE of *C. diphtheriae*, each of which is also homologous to IucA and IucC, may perform functions in biosynthesis of corynebactin that are comparable to the functions of SfnB and SfnD from *S. aureus* in the biosynthesis of the structurally related siderophore staphyloferrin A.

Interestingly, our further analysis of CiuE revealed not only that the repeated N- and C-terminal regions contain sequences homologous to *iucC* and *iucA*, but also that each region contains a sequence immediately downstream from the IucA/IucC-related sequence that exhibits homology to FhuF, a bacterial ferric iron reductase protein involved in ferrioxamine B-dependent iron uptake in *E. coli* [36]. Use of the Conserved Domain Architecture Retrieval Tool (CDART)(http://www.ncbi.nim.hih.gov/Structure/lexington/lexington.cgi) to search for other predicted proteins with tandem repeats of domains that contain both IucA/IucC- and FhuF- related regions identified such candidate proteins in *Corynebacterium* spp., *Acinetobacter* spp., *Streptomyces* spp., *Shewanella* spp., and *Methylobacterium* spp. In addition, both the SfaD/SfnaD and SfaB/SfnaB proteins involved in staphyloferrin A biosynthesis contain an FhuF-related sequence downstream from the IucA/IucC-related sequence. Additional studies will be required to determine whether the FhuF-related motifs in CiuE from *C. diphtheriae* function by reducing iron in intracellular ferric-corynebactin complexes, thereby permitting release of ferrous iron and re-cycling of corynebactin to the extracellular milieu to participate further in siderophore-dependent iron uptake.

In summary, we have determined the structure of corynebactin from *C. diphtheriae*, shown that it contains two citrate residues linked by amide bonds to a single lysine residue, and demonstrated that it exhibits biological activity as a siderophore in *C. diphtheriae*. Although the structure of corynebactin has similarities to staphyloferrin A and rhizoferrin, it is a novel structure that, to the best of our knowledge, has not previously been reported for any other siderophore.

## Materials and Methods

### Bacterial strains

*C. diphtheriae* C7(β) [37] was routinely used as the wild-type strain for comparison in ^55^Fe ^3+^ uptake assays. *C. diphtheriae* strain C7(β) Δ*dtxR* [24] was used for production of corynebactin. The siderophore deficient strain *C. diphtheriae* strain C7(−) Δ*ciuE*, [18] which has an in-frame deletion corresponding to 94% of the *ciuE* gene, was kindly provided by Michael P. Schmitt and was used routinely as an indicator strain for detection of corynebactin in growth stimulation assays and in iron uptake assays. We used *in vitro* methods to construct *C. diphtheriae* Δ*ciuA*, which is a strain deficient in ferric-siderophore uptake. To generate an in-frame deletion corresponding to 60% of the coding region of *ciuA*, we used a first primer pair *ciuA*-up (5′-CAAGAACTCCATGCTGCTGC-3′) and *ciuA-*delR (5′-ACCAACCGATCAGTGCGGTGTTGTCAGAGGAACATCCAGC-3′) and a second primer pair *ciuA*-down (5′-GCCCCAATAGCCATGCC-3′) and *ciuA*-delF (5′- GCTGGATGTTCCTCTGACAACACCGCACTGATCGGTTGGT-3′) plus chromosomal DNA from *C. diphtheriae* C7(β) in separate PCR reactions to produce DNA fragments consisting of approximately 1000 bp from the upstream and downstream flanking regions of *ciuA* plus short segments from the contiguous 5′ and 3′ ends of *ciuA* gene, respectively. These amplicons were used as templates in a second PCR reaction with primers *ciuA-*up *and ciuA*-down to generate a Δ*ciuA*, similar to a previously described method for generating in-frame deletions in the alanine racemase genes of *B. pseudomallei* and *B. mallei* [38]. The resulting Δ*ciuA* amplicon was cloned into pCR2.1-TOPO® (Invitrogen, Carlsbad, CA), resulting in pCR2.1-TOPO® Δ*ciuA*. The pCR2.1-TOPO® Δ*ciuA* clone was digested with *Hind*III and *Xba*I, and the fragment containing Δ*ciuA* was ligated into *Hind*III- and *Xba*I-digested pK19mobsacB [39] to produce pK19mobsacBΔ*ciuA*. The pK19mobsacBΔ*ciuA* construct was introduced into the chromosome of *C. diphtheriae* C7(β) by conjugation, and the co-integrants were resolved by counter-selection with sucrose, as previously described [24]. PCR tests on individual resolved colonies were used to identify *C. diphtheriae* C7(β) Δ*ciuA* and distinguish it from wild type *C. diphtheriae* C7(β).

### Production and detection of corynebactin

The corynebactin over-producing strain *C. diphtheriae* C7(β) Δ*dtxR*, was grown overnight in two 500 ml aliquots of low-iron PGT maltose medium [24] in separate 2.8 l Fernbach flasks. The cultures were centrifuged to pellet the bacteria, and the supernatants were collected. Corynebactin was detected in culture supernatants or in fractions collected from chromatographic procedures using a modified version of the Chrome Azurol S (CAS) assay as described previously [24]. A standard curve was generated using ethylenediamine-*N*,*N*-diacetic acid (EDDA) at concentrations from 1 to 40 µM. One siderophore unit had the same activity in the CAS assay as a control performed with a 0.1 ml sample of 1 µM EDDA [24].

### Purification of corynebactin

Following centrifugation of the culture and subsequent filtration of the supernatant to remove any residual bacteria, the supernatants from cultures of *C. diphtheriae* strain C7(β) Δ*dtxR* [24]were mixed with 48 g of ion exchange resin AG1X2 and allowed to stand for 2 h at room temperature to allow corynebactin to bind to the resin. All of the following procedures were performed at room temperature. The AG1X2 resin was transferred to a gravity flow column and washed sequentially with 1 l of 2.5% ammonium acetate, pH6.5, followed by 0.5 l of 6% ammonium acetate, pH 6.5. Previous experiments showed that most of the corynebactin was eluted with 6% ammonium acetate, pH 6.5, and little additional corynebactin was recovered by a subsequent wash with 8.0% ammonium acetate, pH 6.5. Fractions were collected during the wash with 6% ammonium acetate, pH 6.5, and the fractions with CAS activity were pooled, lyophilized, and dissolved in a sufficient volume of deionized water to decrease the final conductivity to less than 40 mS/cm. The resulting solution (200 ml) was loaded onto a 10 mm×580 mm Source15Q ion exchange column (GE Healthcare, Pittsburgh, PA) equilibrated with 2.5% ammonium acetate, pH 6.5, on an ÄKTApurifier™ 10 (GE Healthcare, Pittsburgh, PA). The column was washed with two column volumes (CV) of 2.5% ammonium acetate, pH 6.5, eluted with a 10-CV linear gradient from 2.5% to 7.5% ammonium acetate, pH 6.5, and subjected to a final wash with 15% ammonium acetate, pH 6.5. Fractions were collected, and quantitative CAS assays for siderophore were performed on each fraction. Fractions with siderophore activity were pooled and lyophilized.

Appropriate conditions for further purification of corynebactin by RP-HPLC on Zorbax C8 matrix (Agilent Technologies, Colorado Springs, CO) were determined empirically. We dissolved the lyophilized corynebactin from the previous Source 15Q purification step in 15 ml of distilled water, adjusted the solution to pH 3.1 by drop-wise addition of formic acid, and loaded the sample onto a 9.4 mm×250 mm, 5 µm Zorbax C8-RP-HPLC column equilibrated with Buffer A (0.6% formic acid) on an ÄKTApurifier™ 10 (GE Healthcare, Pittsburgh, PA). After washing the column with 2 CV of Buffer A, we eluted the corynebactin a 20 CV linear gradient from Buffer A to Buffer B (0.6% formic acid, 80% acetonitrile) at a flow rate of 2 ml/min. Each fraction was tested for CAS activity, and the fraction with peak CAS activity had a retention time of 20.37 min. The fractions with CAS activity were pooled, lyophilized and resuspended in H_2_O for subsequent experiments.

### Biological activity of corynebactin

Fractions collected during each stage of purification were tested for biological activity. Briefly, *C. diphtheriae* strain C7(−) Δ*ciuE* [18] was grown in an 80∶20 mixture of PGT medium (with 10 g Casamino acids/L) and CHI broth (chelexed heart infusion broth) for 18 hours. *C. diphtheriae* C7(−) Δ*ciuE* (at a final density of about 10^6^ bacteria/ml) was added to molten HIBTW agar media (consisting of heart infusion broth, 0.2% Tween 80, ≥60 µM EDDA to chelate iron, and 1.5% g of noble agar/l) at 45°C, and the inoculated molten medium was rapidly poured into petri dishes. After the agar solidified, a 10 ml overlay of 1% agarose containing 0.01% triphenyltetrazolium chloride was added to each plate, and wells were cut into the agar plates by excising plugs with a sterile plastic Pasteur pipette. Samples containing 25 µl of each sample to be tested for siderophore activity, with and without FeCl_3_ at a final concentration of 25 µM, were added to the wells. Growth stimulation was assessed after incubating the plates for 18 h at 37°C by measuring the diameters of the red zones produced by reduction of the triphenyltetrazolium chloride dye as a consequence of growth of the bacteria around the wells. To determine the quantitative relationship between CAS activity and biological activity of corynebactin, a sample of purified corynebactin with CAS activity equivalent to 108 µM EDDA was diluted in a two-fold series ending at 0.875 µM EDDA equivalents, and samples of each dilution were tested in triplicate in the bioassay described above along with a control that had zero corynebactin. The average diameters of the growth stimulation zones were measured and plotted against the CAS units of corynebactin added to the wells in the bioassay plates (see Fig. 2B).

### Stimulation of iron uptake in *C. diphtheriae* by corynebactin

^55^Fe^3+^ uptake assays were performed as previously described [18]. Control experiments were used to determine the ability of wild-type and mutant strains of *C. diphtheriae* to take up ^55^Fe^3+^ in the absence of added corynebactin. Briefly, overnight cultures of C7(β) (wild-type strain), C7(−) Δ*ciuE* (siderophore deficient strain), and C7(β) Δ*ciuA* (deficient in utilization of the ferric-siderophore complex) were subcultured into 80∶20 PGT∶CHI medium described above and grown to an OD_600_ = 0.5–0.8. Uptake assays were initiated by adding 0.1 µCi ^55^Fe^3+^, and 50 µl aliquots were removed from the cultures and applied to nitrocellulose filters (0.45 µm pore size) under vacuum at times 0, 1, 2.5, 5, 10, and 20 min. Immediately after addition of the culture sample, each filter was washed twice with 100 µl volumes of 100 mM sodium citrate, and the amount of ^55^Fe^3+^ assimilated by the bacteria was measured using a liquid scintillation counter. To assess the ability of purified corynebactin to promote iron transport in each of the strains of *C. diphtheriae* described above, samples of each culture were centrifuged to collect the bacteria, the supernatants were removed, each bacterial pellet was suspended in a volume of fresh medium equivalent to the original size of the culture sample, purified corynebactin was added to each sample at a final concentration of 4 µM, and iron uptake assays were initiated by addition of ^55^Fe^3+^ as described above. All assays were performed in triplicate, and standard deviations of the mean were calculated for each set of assays.

### NMR analysis

All NMR experiments were performed on a 500 MHz Varian Inova spectrometer equipped with a 5 mm triple resonance HCN probe with Z-axis pulsed field gradients. All experiments were recorded at 25°C. Samples were dissolved in 90% H_2_O/10% D_2_O or 100% D_2_O and 4,4-dimethyl-4-silapentane-1-sulfonic acid (DSS) was added to a final concentration of ∼0.05% as a chemical shift reference. Carbon and nitrogen chemical shifts were referenced using indirect chemical shift ratios to calculate the frequencies at 0 ppm for the respective nuclei [40].

A DQF-COSY [41], [42] spectrum of a sample dissolved in 90% H_2_O was recorded using 4096 (t2)×400 (t1) complex data points and with spectral widths of 6000 Hz in both dimensions. A ^1^H- ^1^H NOESY spectrum [43] was recorded using 4096 (t2)×300 (t1) complex data points and spectral widths of 6000 Hz in both dimensions. A ^1^H-^13^C Heteronuclear Single Quantum Correlation (HSQC) spectrum [44], [45] was recorded with 4096 (t2)×300 (t1) complex data points and with spectral widths of 6000 Hz (F2) and 6400 Hz (F1). A ^1^H-^13^C Heteronuclear Multiple Bond Correlation (HMBC) experiment was recorded using 4096(t2)×1024 (t1) complex data points with spectral widths of 4000 Hz (F2) and 26,000 Hz (F1). A ^1^H-^15^N HSQC spectrum was recorded with 2048 (t2)×128 (t1) complex data points and with spectral widths of 6000 Hz (F2) and 2200 Hz (F1). In all cases, the initial value of the incremented delay was set to one-half of the dwell time in the indirectly detected dimension. Quadrature detection in the indirect dimension was achieved using the States-TPPI method [46]. For experiments recorded in 90% H_2_O, water suppression was achieved by pre-saturation of the solvent signal. For DQF-COSY experiments recorded at pH 4, solvent suppression included saturation of the solvent during the incremented evolution time. This introduces a Bloch-Siegert shift in the position of resonances close to the water signal. This shift is not present in any of the other spectra and all reported chemical shifts are reported relative to these unshifted signals at pH 6.0.

All spectra were processed using NMRPipe [47]. The raw data were multiplied by a sine bell function shifted by 90°, followed by multiplication by a mild Lorenz-Gaussian function. The data were zero filled once in the directly detected dimension (t2) and twice in the indirect dimension (t1) prior to Fourier transformation. HMBC experiments were visualized in absolute value mode. NMR spectra were analyzed using Sparky [48].

### Electrospray ionization mass spectrometry

Electrospray mass spectra were collected on a QSTAR XL (Applied Biosystems, Foster City, CA) operating in negative ion mode. The instrument was fitted with a NanoSpray ionization source (Applied Biosystems, Foster City, CA). Sample was introduced into the ionization source at a flow rate of 500 nl/min via a syringe pump (KD Scientific, Holliston, MA) and sprayed through a glass capillary with a tip diameter of 15 µm (New Objective, Woburn, MA). Reported *m/z* values are the average of five separate mass spectra. Mass spectra of purified corynebactin from *C. diphtheriae* were externally calibrated against a commercially available sample of rhizoferrin purchased from EMC Microcollections (Tuebingen, Germany).

### Chemical assays for siderophores

Samples of purified corynebactin were hydrolyzed with acid according to published procedures cited below and tested to detect the presence of catechols, hydroxamates, citric acid, or amino acids.

To test for catechols, we performed the Arnow assay [49]. We purified a sample of bacillibactin from *Bacillus subtilis* [20] according to a protocol provided by Sandra Armstrong at the University of Minnesota and used the purified bacillibactin as a positive control for the Arnow assay. We used the Csaky assay to test for hydroxamates [50]. Both hydrolyzed and unhydrolyzed samples were tested in the Csaky assay, and desferioxamine (Sigma-Aldrich, St. Louis, MO) was used as a positive control.

To test for citric acid, purified corynebactin was hydrolyzed in 6 M HCl and heated for at least 8 hours at 100°C. The amount of citric acid in an acid hydrolyzed sample of purified corynebactin was measured with the Boehringer Mannheim/R-Biopharm Citric Acid UV Method kit using instructions provided by the manufacturer (Roche, Darmstadt). Briefly, 1 ml of solution 1 (containing glycylglycine buffer pH 7.8; L-malate dehydrogenase, L-lactate dehydrogenase, and NADH) was mixed with 0.2 ml of hydrolyzed corynebactin diluted to 1 ml in double distilled H_2_O. After 5 min of incubation, the absorbance was read at 340 nm and denoted as A_1_. The reaction was then initiated by adding solution 2 (containing citrate lyase), and after 5 min of incubation the absorbance was measured again (A_2_). The change in absorbance (A_1_–A_2_) was used to calculate the concentration of citric acid in the sample.

To test for amino acids, a sample of purified corynebactin was hydrolyzed in 6 M HCl (also containing 0.1% phenol) at 110°C for 24 h in sealed and evacuated tubes. Analysis was carried out on a Beckman System 6300 Amino Acid Analyzer (Beckman, San Ramon, CA), the separation mechanism of which involves cation-exchange chromatography and post-column ninhydrin derivatization. Amino acids or peptides are eluted based on net charge and hydrophobicity. Runs consisted of a series of three buffers: 0.2 M sodium citrate, pH 3; 0.2 M sodium citrate, pH 4.3; and 0.35 M sodium citrate, 0.75 M NaCl, pH 7.9. Eluted samples were mixed with ninhydrin reagent and detected at 570 nm for primary amines and 440 nm for secondary amines.

## Supporting Information

Figure S1 **Initial purification of corynebactin by anionic exchange chromatography on Source 15Q resin.** Corynebactin was recovered as a single activity peak at approximately 6% ammonium acetate during elution with a 2.5% to 7.5% linear gradient of ammonium acetate. Absorbance at 210 nm (A_210_) is shown as a partially dashed line; the linear ammonium acetate gradient followed by a 15% ammonium acetate wash is shown as a continuous solid line; and CAS activity is shown as a dotted line.
(TIF)
