# Supplementary figures and images for: Purification and Structural Characterization of Siderophore (Corynebactin) from Corynebacterium diphtheriae
Source: PLoS One. 2012 Apr 13;7(4):e34591. doi: 10.1371/journal.pone.0034591 (PMC3326035; doi:10.1371/journal.pone.0034591)

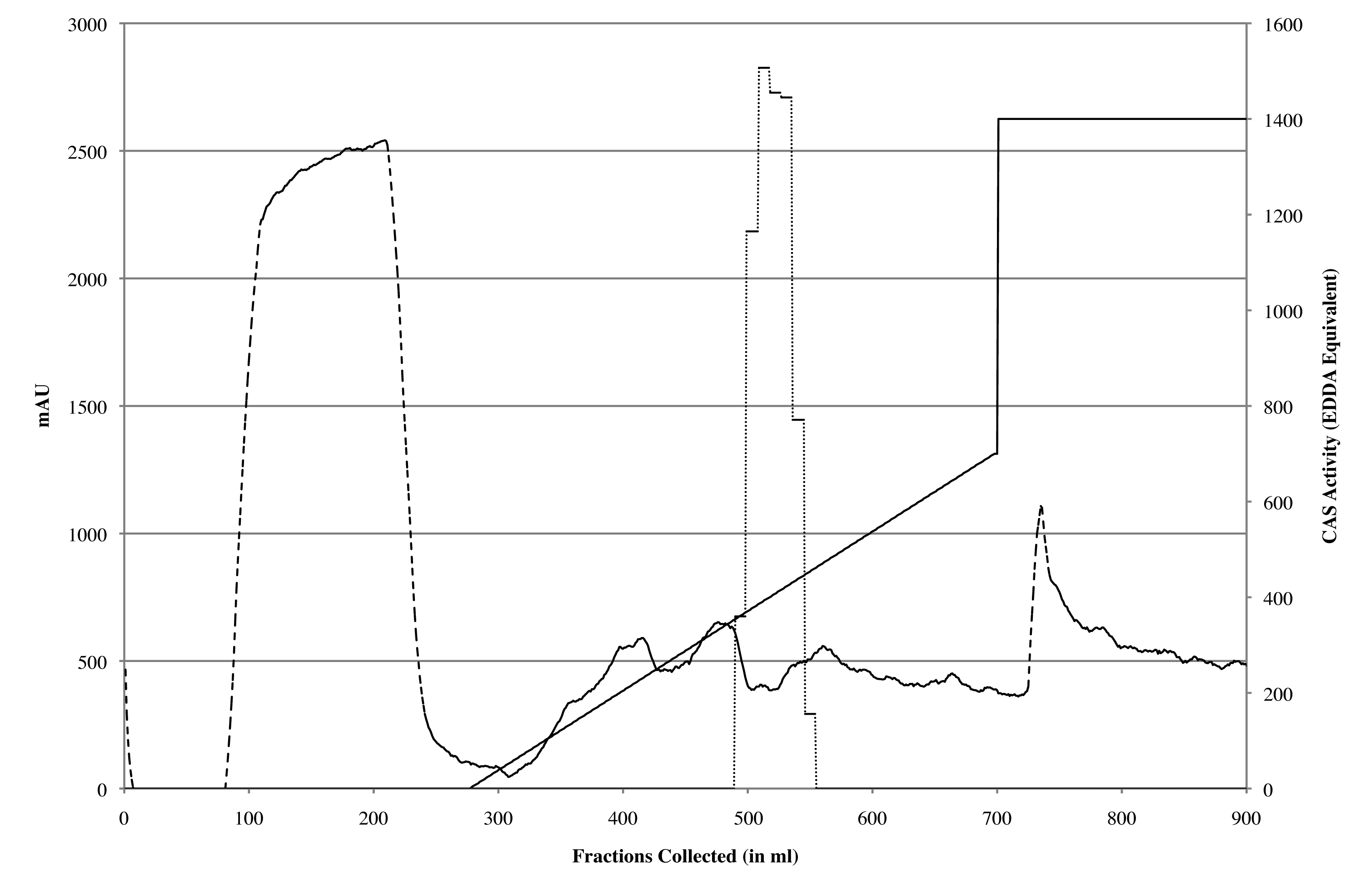

Supplement: Figure S1 — Initial purification of corynebactin by anionic exchange chromatography on Source 15Q resin. Corynebactin was recovered as a single activity peak at approximately 6% ammonium acetate during elution with a 2.5% to 7.5% linear gradient of ammonium acetate. Absorbance at 210 nm (A210) is shown as a partially dashed line; the linear ammonium acetate gradient followed by a 15% ammonium acetate wash is shown as a continuous solid line; and CAS activity is shown as a dotted line. (TIF) [file pone.0034591.s001.tif]
